# Gender affirmative HIV care framework: Decisions on feminizing hormone therapy (FHT) and antiretroviral therapy (ART) among transgender women

**DOI:** 10.1371/journal.pone.0224133

**Published:** 2019-10-21

**Authors:** Arjee J. Restar, E. Karina Santamaria, Alexander Adia, Jennifer Nazareno, Randolph Chan, Mark Lurie, Theo Sandfort, Laufred Hernandez, Susan Cu-Uvin, Don Operario

**Affiliations:** 1 Department of Behavioral and Social Sciences, Brown University School of Public Health, Providence, RI, United States of America; 2 The Philippine Health Initiative for Research, Service, and Training, Brown University School of Public Health, Providence, RI, United States of America; 3 amfAR, The Foundation of AIDS Research, Washington, DC, United States of America; 4 Department of Special Education and Counselling, The Education University of Hong Kong, Tai Po, Hong Kong; 5 HIV Center for Clinical and Behavioral Studies, Department of Psychiatry, Division on Gender, Sexuality, and Health, New York State Psychiatric Institute and Columbia University, New York, NY, United States of America; 6 Department of Behavioral Sciences, University of Philippines in Manila, Manila, Philippines; 7 Providence-Boston Center for AIDS Research, Providence, RI, United States of America; 8 Miriam Hospital, Department of Medicine, Providence, RI, United States of America; Ruprecht Karls University Heidelberg, GERMANY

## Abstract

**Background:**

Integration of feminizing hormone therapy (FHT) and antiretroviral therapy (ART) is critical in providing gender-affirming HIV care for transgender (trans) women living with HIV. However, interpersonal communications with HIV providers who are not competent with FHT may complicate this integration.

**Methods:**

We conducted semi-structured interviews with trans women (n = 9) who self-reported as HIV-positive and their HIV providers (n = 15) from community-based venues (e.g., clinics) in Manila, Philippines.

**Results:**

We identified five key themes from our qualitative data: (1) provider’s concerns; (2) patient’s goals; (3) affirmative vs. non-affirmative provider rhetoric; (4) alignment vs. misalignment of provider rhetoric to patient goals; and (5) FHT and ART-related decisions. Based on these themes, we describe a gender-affirmative HIV care framework to understand FHT-ART decisions among trans women living with HIV. Based on our data, this framework shows that provider-patient communications regarding ART and FHT consists primarily of provider concerns and patient goals regarding FHT. These communications can take on a gender-affirmative or non-affirmative style of rhetoric that either aligns or misaligns with patient goals and may lead to differences in FHT and ART-related decisions among trans women living with HIV.

**Conclusion:**

There exist mixed regimens and beliefs about taking FHT and ART among this sample of trans women. While trans participants’ main source of health information is their HIV provider, providers are likely to communicate non-affirmative rhetoric that negatively impacts trans women’s decision to take FHT and ART. Research is needed to elucidate co-prescriptions of gender-affirmative services with HIV care among this group in the Philippines.

## Introduction

Globally, transgender women (trans women) are communities of women disproportionately burdened by HIV [1–5]. A global meta-analysis of research on HIV infection among trans women showed a prevalence of 19% (95% confidence interval = 17% to 21%) [1]; when compared to all cisgender (non-transgender) adults of reproductive age, adult trans women have a 49-fold increased odds of being HIV infected [1]. The Philippines has the fastest-growing HIV epidemic in the Asia-Pacific region with a 141% increase from 2010 to 2016 [6, 7]. UNAIDS has declared HIV a national public health crisis in the Philippines [2, 4, 8]. In 2017, there were 11,103 reported new HIV cases in this country, which is a 20% increase from the 9264 new cases reported in 2016 [9]. These significant increases in new HIV cases in the Philippines have been concentrated in a few key affected populations including trans women [2].

The Philippines’ 2015 Integrated HIV Behavioral and Serologic Surveillance (IHBSS) Report shows that among Filipina trans women surveyed (n = 3535), nearly 40% took hormones by pills and injection in the past year, and 40% had feminizing surgical body enhancements [10]. Additionally, 38% of trans women in two major Philippines cities demonstrated a desire for sexual reassignment surgery. Among trans women living with HIV, however, only 1% report being on antiretroviral therapy (ART), and significant drops have also been reported along the HIV care continuum leading to low levels of adherence and viral suppression [10].

In the context of providing HIV care and treatment among trans women living with HIV, ‘gender affirmative care’ refers to the process where trans women receive necessary HIV care and treatment services which also ensure medical, social, structural recognition and support for their gender identity and expression [11–13]. Gender-affirmative care is multifaceted and includes the critical integration of both HIV care and treatment services with gender-affirming health services [14, 15]. Research suggests that the provision of and access to feminizing hormone therapy (FHT) and ART *combined with* trans affirmative care are vital for improving engagement and retention and for achieving viral suppression among this population [3, 15–17].

Previous studies with trans women have shown that adherence to FHT and experience of gender affirmation lead to higher ART adherence [14, 15, 18] and viral suppression [14, 19, 20]. Additionally, a key interpersonal and social aspect of gender-affirmative care includes having trained HIV providers who are competent in delivering ART and FHT services to trans women and who are affirming of trans women’s gender and womanhood during patient-provider interactions [12, 21]. These factors have shown to increase positive healthcare experiences and engagement in HIV care and treatment [12, 14, 20, 22, 23]. Moreover, structural factors like healthcare provider trainings that support ART and FHT prescribing practices and services for trans women also enhance healthcare facility-level norms and policies around gender-affirmative care [14, 15, 17, 20]. As such, gender-affirmative care for trans women highlight the importance of integrating and facilitating HIV care and treatment services with gender-affirming services like FHT in order to be respectful of the health needs of trans women [22].

However, availability of HIV facilities and providers who offer gender-affirmative care in the Philippines are limited, presenting an accessibility issue for trans women living with HIV. In the metropolitan city of Manila where HIV is highly concentrated [2], there are 14 HIV Counselling and Testing facilities as of 2018, of which none are known to provide FHT or other health services tailored to Filipina trans women [5]. The Philippines’ health system, research priorities, and programmatic response to the HIV epidemic focuses largely on cisgender female sex workers, with few services focusing on men who have sex with men, and no documented services focusing on trans women [7, 24, 25]. Problems with HIV treatment services for trans women in the Philippines are further compounded by the lack of research dedicated to trans women’s HIV treatment and FHT medications at the structural-level, stigma and discrimination with providers at the interpersonal-level, and reported concerns about adverse side-effects between ART and FHT drug interactions at the personal-level [14, 17, 20, 26, 27]. In a recent review, researchers noted that concern for negative side-effects may arise from the dearth of data that address interactions between ART and FHT among trans women [26].

Moreover, perspectives on and utilization of both FHT and ART among trans women living with HIV are not well-documented in the Philippines. Research on the relevant knowledge and skills of HIV providers in the Philippines in also limited [24]. While it is known that trans women living with HIV face many socio-ecological barriers that negatively impact uptake, retention, and adherence to HIV treatment services [14, 23, 28], far less is known about their decision-making process for ART and FHT in the presence or absence of gender-affirmative care.

Given the potential for gender-affirmative care to facilitate trans women’s linkage, retention, and adherence to HIV treatment and the unmet HIV health needs of this population in the Philippines, this exploratory study sought to examine the dynamics of ART and FHT decisions in the context of gender-affirmative care. We examined interviews with trans women living with HIV and with HIV providers to discuss (1) views and experiences with ART and FHT from both the patient and provider perspectives, and (2) the potential role HIV providers’ incorporation of gender-affirmative care in shaping views and decisions related to ART and FHT among trans women.

## Methods

### Research team

S1 Table displays the Consolidated criteria for Reporting Qualitative research (COREQ) Checklist [29]. The interview team on site in Manila, Philippines consisted of a total of three interviewers, who are all graduate-level Filipina-Americans and are trained in qualitative interviews, including the corresponding lead author (AR) who is fluent in Tagalog. The research study team is composed of US- and Philippines-based researchers, as well as two HIV non-governmental organizations (NGOs) in Manila which served as our local research collaborators for this study. The study team had no prior relationships with participants prior to the study commencement.

### Study design

Using a phenomenological approach to qualitative investigation [30], we sought to explore and deepen our understanding about the dynamics of ART and FHT decisions in the context of gender-affirmative care among trans women who are living with HIV as participants, and their healthcare providers as key informants, in Manila, Philippines. We utilized semi-structured, one-on-one in-person interviews to gather qualitative data until saturation was reached with regard to our primary aims [31].

We sampled participants using passive and active recruitment approaches. Passive recruitment strategy included posting study flyers at venues where trans women congregate such as HIV clinics, non-profit organizations, and community centers. Active recruitment included using snowball sampling technique [32], in which we asked participants to refer other potential participants. Given that this community is highly stigmatized in the Philippines, these recruitment approaches were deemed appropriate by our local study partners. Key informants were recruited via referrals from HIV NGOs. Given that there is no publicly available dataset of HIV providers in the Philippines, we recruited them as key informants via snowball sampling. Our local study partners who have deep knowledge of the local HIV NGOs in Metro Manila identified sites to recruit key informants. Invitations to participate in the study were extended to these sites.

All procedures performed in this study were in accordance with the ethical standards of the institutional research committee (Brown University Ethics Review Board, IRB protocol #1705001780) and with the 1964 Helsinki declaration and its later amendments or comparable ethical standards. Informed consent was obtained from all individual participants included in the study.

### Participants and key informants

A total sample of 24 participants were included in this study (9 trans women and 15 HIV care providers). Trans women were eligible if they: (1) were 18 years of age or older; (2) self-identified as trans women; (3) self-reported an HIV positive status; (4) reported any HIV-related behavioral risk in the past year with a male sexual partner; (5) resided in Metro Manila area; (6) were willing to be audio-recorded; and (7) were willing to provide written informed consent. We also included HIV healthcare providers from the Metro Manila area. Healthcare providers were eligible if they: (1) were 18 years or older, (2) currently working in a facility that offers HIV/AIDS services, (3) have served trans women as patients (e.g., interacted with and provided HIV-related care services for trans women), and (4) were willing to be audio-recorded and provide written informed consent. The definition of healthcare providers was broad such as to include medical doctors, nurses, and medical clinic coordinator / counselor, and staff volunteer. This broad definition was recommended by our local study partners to ensure that the inclusion of HIV NGOs and health centers, particularly those which do not have medical doctors but provide HIV care to trans women, were reflected in our sample.

### Procedures

Participants were screened by telephone and were scheduled for an in-person interview. Interviews lasted between 60 to 90 minutes and were conducted in a private place at the local offices of our study partners. Interviews were conducted in English. Because English is one of two primary languages besides Tagalog and is integrated throughout the Philippines’ education system [33], language was not a barrier to recruitment or to building rapport with participants.

Between July and August 2017, participants and key informants were interviewed for the study by a trained interviewer using a semi-structured interview guide. The purpose of the interview guide for trans women participants was to understand the personal, social, and structural contextual factors that might influence their behaviors around uptake of HIV treatment services like ART. Specifically, participants were asked about their history of taking FHT and ART medicines, their knowledge and experiences about taking these medications, their concerns, if any, about negative side effects or drug-drug interactions, where and what kind of information they receive about FHT and ART, and how they resolve information that may be in conflict of their gender-affirming goals Key informants were asked about similar issues in order to create a comprehensive understanding about how trans women may utilize HIV services with FHT. Specifically, key informants were asked about the provision of HIV care to trans women, their experiences providing care to trans women, and how they approach the use of FHT and ART. Interview guides were piloted with two trans women community members and two key informants from our local HIV NGOs study partners.

Written informed consent was collected from all participants prior to interviews. During the informed consent process and prior to enrollment to the study, participants were provided information about the research study including the purpose of the study, and their rights to confidentiality, withdrawal, and refusal to answer any questions. Besides name of the interviewer, there were no other interviewer characteristics provided to participants that could bias the interviews. Trans women and health care providers who completed the interview were compensated 500 Philippines Pesos (~$10). All study procedures were approved by Brown University Ethics Review Board.

### Analysis strategy

**Fig 1:**
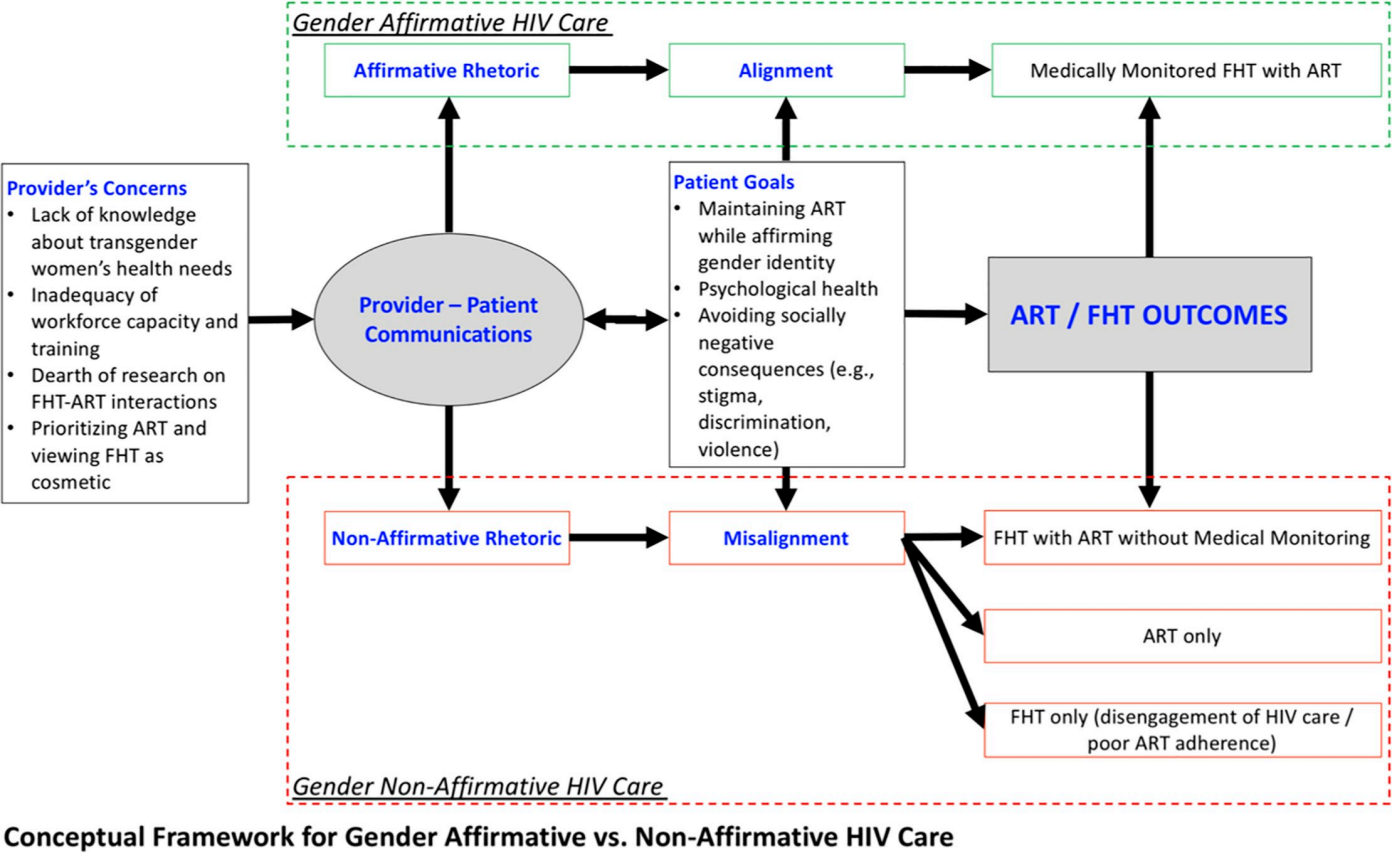
Conceptual framework for gender affirmative vs. Non-affirmative HIV care.

Guided by Braun and Clarke’s thematic analysis approach [34], four members of the study team participated in developing a codebook that facilitated identification of emergent themes. All research members were trained in the application of the codes. Any discrepancies in coding were discussed via research meetings until consensus was reached. For this study, we utilized the following codes from participants’ perspective: (a) personal experiences with taking ART, (b) interactions and discussions of ART with healthcare providers, (c) motivations, experiences, and issues around taking FHT, and (d) interactions and discussions of FHT with healthcare providers. We utilized the following codes from key informants’ perspective: (a) FHT awareness, knowledge, and views, (b) ART awareness, knowledge, and views, and (c) Trans service delivery challenges and solutions. Emergent and final themes were agreed upon by all members of the study team. All coding and analysis were performed using an online qualitative data management software, Dedoose [35]. Following the coding of both sets of interviews, three members of the study team triangulated interviews of key informants and trans women to build themes regarding ART and FHT [36]. Based on identification and interpretation of themes, members of the study developed a working conceptual framework for understanding the processes related to gender-affirmative care around the use of FHT and ART (see Fig 1).

## Results

### Sample characteristics

Summary of provider and patient demographics are displayed in Table 1. On average, trans women in this sample were 25.6 years of age. The majority had attained at least a college education. Providers’ characteristics varied by role/title, type of facility they work in. The sample consisted of two providers working at public hospitals, three at social hygiene clinics, eight at HIV NGOs, and two at state-sponsored HIV organizations.

**Table 1:** Demographic characteristics of transgender women (n = 9) by age, education, and HIV status, and healthcare providers (n = 15) by role/title, and type of facility.

Trans women’s characteristic (n = 10)	Count
Age	
Mean (SD, range)	25.0 (3.1, 18–29)
Education	
High school or less	4
College or more	5
Self-reported HIV status	
Positive	9
Negative	0
**Provider’s characteristics (n = 15)**	
Type of facility providers work in	
Public Hospital	2
Social Hygiene Clinic	3
Non-profit HIV Organization	8
State-sponsored HIV Organization	2

### Overview of key concepts

Five key themes emerged from our qualitative data analysis: (1) provider’s concerns; (2) patient’s goals; (3) affirmative vs. non-affirmative rhetoric; (4) alignment vs. misalignment of rhetoric to patient goals; and (5) FHT and ART related decisions. These five themes are illustrated below via quotes from trans women and their HIV healthcare providers. They are also explained and further interpreted in the discussion section and in Fig 1. In Fig 1, we present a preliminary conceptual framework of gender-affirming HIV care based on these findings. Our model shows that provider-patient communications regarding FHT and ART consists primarily of provider’s concerns and patient goals regarding FHT. These communications can take on a gender-affirmative or non-affirmative rhetoric style that either aligns or misaligns with patient goals and may lead to differences in FHT and ART related decisions among trans women.

### Provider’s concerns

When asked about their thoughts regarding the utilization of FHT and ART medications among trans women living with HIV, most HIV providers expressed the following concerns:

#### (1) Lack of knowledge about transgender women’s health needs

Most providers expressed awareness and “superficial” knowledge about trans women’s gender affirmation health needs including FHT. As one provider illustrated:

“[Hormone] is a very popular thing to do—it's kinda expensive, the [hormone] medical management. That's my impression…How, what happened and what are the physical changes and what medications—it's more a mystery thing. But there are also resources on the psychological impact of this kind of patient. More or less, we have superficial [knowledge], but the awareness is there.” (Provider 3, social hygiene clinic)

#### (2) Inadequacy of workforce capacity and training in gender-affirmative HIV care

Other providers expressed concerns towards FHT as a service that is outside the purview of their clinic/hospital’s general care. As FHT is not offered in their practice, providers are unsure about their staffs’ capacity and readiness given their inadequate training to “handle” trans women’s gender affirmation health needs. As these providers described:

“I'm not aware that we have a service in the charity or outpatient department for that…it's a general hospital so maybe we have plastic surgery, but we don't have any cases like that… I don't know if an ordinary resident or nurse can handle that [providing FHT] …and training for TGs [transgender people] no, we don't have that. It's general.” (Provider 3, social hygiene clinic)

“[The] facilities are not yet ready for [FHT]—I'm also not sure or aware if there are any services available in the country. Within our network, I don't think that we have that in place.” (Provider 14, state-sponsored HIV organization)

#### (3) Dearth of research on FHT-ART interaction and beliefs about potential side effects

Providers noted that there are no FHT-ART studies in the context of Filipina trans women. Other providers who are familiar with the medical research literature noted the scant evidence around FHT-ART interactions. This dearth of research influences their beliefs and hesitations to provide both FHT and ART medications to Filipina trans women living with HIV. As one provider stated:

“We lack data in terms of [whether there is] contradiction in taking FHT and ART. It’s the same issue for taking FHT and ART if you’re a trans PLHIV [person living with HIV]. Those kinds of research are not available. We have international studies in the [United] States that say there is none, no contra indication, but of course it affects your liver, your lipid profile, and because you’re taking too much different kinds of meds in your body.” (Provider 11, HIV NGO)

#### (4) Prioritizing ART and viewing FHT as cosmetic and unimportant

Some HIV providers perceived ART as more life-saving than FHT, viewing FHT as a cosmetic need to make trans women’s skin “fair:”

“I think the challenge would lie on the use of ARV coupled with the use of other drugs, like hormones—which is used to make their skin fair.” (Provider 14, state-sponsored organization)

It was also common for providers to suggest that trans women should stop taking FHT to allow ART to work in the body without any contradiction or side effects from FHT, like this provider explained:

“Hmm, the FHT. That's maybe not so possible here in [this hospital] for now. The FHT vs. ART, that's my main problem. Because we do client [who are] trans PLHIV [person living with HIV], they are so very sad [we] tell them to stop taking FHT…it's maybe the contradiction between FHT and ART. The side effects for, so that's why [we] want [them] to stop FHT.” (Provider 1, public hospital)

This prioritization of ART over FHT is evident from another provider’s excerpt who mentioned how they do not “focus on what [trans women] are talking about” when it comes to the “pills they are taking.” As this provider described:

“What would be their issues? I don't know, I don't have much idea about the needs of TGs [transgender people]. I know—I heard from people have this support group here, TGs are taking medications for hormones. I do not know that. I just don't focus on what they are talking about, the pills they are taking, you know?” (Provider 10, HIV NGO)

### Patient’s goals

All trans women in this sample expressed their goals and reasons for using FHT in the context of living with HIV. These goals include continuing to take ART while having their gender affirmed, attaining self-confidence and self-esteem, and avoiding violence, as described below:

#### (1) Maintaining ART medication while affirming their transgender identity, femininity, and womanhood

Trans women expressed that taking FHT enables them to be themselves–as women. One trans woman mentioned that taking hormones was their “biggest dream” as it is part of their gender affirmation goals. Another trans woman expressed that they wanted to maintain taking ART while retaining their womanhood and continuing to live their life as a woman. As they explained:

“Well, taking hormones is my biggest dream. For me to have breasts, a more girly voice.” (Participant 2, age 29)

“We want to look woman, we want to look feminine. And we want to live life with ART.” (Participant 1, age 26)

#### (2) Psychological health

Other trans women living with HIV disclosed that in addition to facilitating their gender goals, taking FHT also improves their self-confidence and self-esteem. Specifically, one trans woman noted that FHT “boost [their] system” and how they feel about themselves as woman. This suggests that trans women see the importance of taking FHT as not only about attaining their desire look physically, but that FHT also provides mental health benefits, including a sense of confidence about who they are and of viewing themselves as beautiful. As they described:

“[If] you're taking feminizing hormones, you will look like feminine. So it will boost your system” (Participant 1, age 26)

“P: When I take it, it's too important because it's feels like you're fresh, you have that glowing …glow, it’s feels like you're beautiful that's why it's important.” (Participant 8, age 25)

#### (3) Avoiding socially negative consequences (e.g., stigma, discrimination, and violence)

In addition to the physical and mental health benefits, some trans women noted how FHT is important for avoiding socially negative consequences when interacting with other people; these interactions can include being stigmatized, discriminated, and being targeted for violence. One trans woman explained how access to FHT is particularly important in the Philippines where simply dressing as a woman is not enough to avoid socially negative consequences; without FHT to help with their gender transition, they are further singled out and prone to violence including being bullied:

“Because being transgender is not only to look feminine. Here in Philippines… if you want to be a woman, first, they want to see you as like a woman. [If you are] appearing [and only] wearing like a woman's dress, they [people] will bully you…if you're not [seen with] a feminine look but you're wearing like a woman, they will tell you you're a cross dresser, you're yuck. You have a hard face.” (Participant 1, age 26)

### Affirmative vs. Non-affirmative rhetoric style from provider-patient communications

Table 2 describes trans women’s interactions with their healthcare providers and their actions in regard to taking FHT with ART. With the exception of two trans women who did not explicitly state their response, all trans women in this study reported discussing taking FHT and ART with their healthcare providers (i.e., provider-patient FHT-ART communications). Five out of nine trans women in the sample reported being advised by their providers to stop taking FHT with ART. When trans women were asked to describe these discussions, our analysis revealed two type or styles of rhetoric that emerged from our qualitative data, affirmative vs. non-affirmative rhetoric styles.

**Table 2:** FHT-ART outcomes of provider-patient communications reported by transgender women participants.

Participant ID	Talked with provider about FHT with ART	Experienced non-affirmative rhetoric with provider	Outcome 1: Medically monitored FHT with ART	Outcome 2: FHT with ART without medical monitoring	Outcome 3: ART only (conformation)
1	Yes	Yes	-	Yes	-
2	Yes	Yes	-	-	Yes
3	Yes	No	Yes	-	-
4	Yes	No	Yes	-	-
5	Yes	No	-	Yes	-
6	Did not state	Did not state	Yes	-	-
7	Yes	Yes	-	-	Yes
8	Yes	Yes	-	-	Yes
9	Yes	Yes	Yes	-	-
**Total (n = 9)**	**Yes = 8**	**Yes = 5**	**Yes = 4**	**Yes = 2**	**Yes = 3**

#### (1) Affirmative rhetoric

In the context of provider-patient communications, affirmative rhetoric are expressions and/or discourse that providers use to specifically convey support for trans women’s goal of taking both ART medication for HIV treatment and FHT for their gender affirmation goals. Providers who utilized this rhetoric emphasized FHT as a crucial element in trans women’s HIV treatment and care and recognized the need for retention in care to monitor pharmacological bodily responses of trans women. In this sample, only two trans women reported that their providers utilized affirmative rhetoric when discussing FHT-ART. One trans woman on hormone-related treatment described an example of affirmative rhetoric by an HIV provider during their conversation when she was first diagnosed with HIV:

“Yes. So, one of the, I mean, the head of the treatment hub or the AIDS research group, our doctor there, so I remember it was my first transition phase, then I was surprised that I was diagnosed with HIV. So, forecasting things that if I may need to [have] ART, I took the liberty of asking that provider, 'Doc, would it be good, would there be any benefit or could there be any contraindication between taking ARTs and hormones?', and I was surprised…that [my doctor said], ‘these are hormones, these are ARTs, you could take them at the same time’ … she told me it would still be best and advised for us to see an endocrinologist. And this way, when the levels our laboratories and stuff, the index are observed, and they are of the safe proportion.” (Participant 3, age 29)

#### (2) Non-affirmative rhetoric

On the other hand, non-affirmative rhetoric prioritized ART over FHT, and neither conveyed encouragement for using FHT nor supported trans women’s gender affirmation goals. Providers who discussed FHT-ART with this style of rhetoric often proscribed or condemned trans women to stop taking their hormones and instead, focus on taking their ART so that they will not “die” from AIDS. One trans woman living with HIV described her discussion experiences with her provider who used non-affirmative rhetoric to discouraged her from taking hormones with ART or else face death as a consequence:

"And the last time I asked about my taking hormones, [the doctors] aren't serious [about] that, they answer me… “Do you want to look feminine? So you want to look feminine, okay. You look feminine, but you will be die…Is that okay to you?" But I have, I have, I have inside of me that you don't understand. Why I want to take hormones. " (Participant 1, age 26)

### Alignment vs. Misalignment of provider rhetoric style with patient goals and FHT-ART outcomes

Half of trans women in this sample reported that they stopped taking FHT after discussions with their health care providers (Table 2). At the time of the interview, only half reported currently taking FHT with ART. Trans women described the type of rhetoric providers used and how such rhetoric aligns or misaligns with their gender affirmation goals, which impacts how they decide to take or not take FHT with ART (i.e., FHT-ART outcomes). Alignment occurs when patient’s goals are supported with provider’s affirmative rhetoric, and misalignment occurs when it is not supported. Three types of outcomes were illustrated by trans women: (1) medically-monitored FHT with ART, (2) FHT with ART without monitoring, (3) ART only.

#### (1) Medically monitored FHT with ART

Trans women with providers who communicated with affirmative rhetoric reported feeling supported to take FHT concurrently with ART as such rhetoric aligned with their gender affirmation goals. A perceived benefit of this was having providers monitor their body’s response to medications and ensure that “they are of the safe proportion.” As one trans woman described:

“[My doctor said], ‘these are hormones, these are ARTs, you could take them at the same time’ … she told me it would still be best and advised for us to see an endocrinologist. And this way, when the levels our laboratories and stuff, the index are observed, and they are of the safe proportion.” (Participant 3, age 29)

#### (2) FHT with ART without medical monitoring

Trans women with providers who communicated with non-affirmative rhetoric frequently described not feeling supported about their gender affirmation goals. One outcome of this misalignment between provider rhetoric and patient goals was that trans women insisted and decided on continuing to take their FHT with ART against their provider’s advice. Because of this misalignment, some trans women decided not to tell their providers about their FHT, which led to an oversight in their care as providers were not able to monitor the effects of hormones in their bodies. As one trans woman and one provider described when asked about her reasons for FHT initiation:

“Because I believe I want to be a female because I was look really masculine before. If ever I'm thinking I want to dress like a girl and I have that muscle in my arms, my legs, I don't feel like a girl then, so I decided, I still decided to take hormones even the medical officers don't want me to take it.” (Participant 10, age 26)

“[It] depends with whether or not the patient would disclose if they are using these [hormones]. So sometimes they would, some patients, they would not…that can be a concern…it's not within our control.” (Provider 14)

#### (3) ART only

Another outcome described by trans women from the misalignment between provider’s non-affirmative rhetoric and patient’s gender affirmation goals is stopping FHT; they conform to their provider’s advice to take ART only. Trans women described that while they had difficulty stopping their FHT, they found themselves in a life vs. death situation and felt pressured to prioritize ART exclusively, which reflects the non-affirmative rhetoric of their providers. Because of how FHT is framed non-affirmatively during their provider-patient discussion, many trans women believed and feared that FHT will impact their ART medication negatively:

“The main reason that I stopped [FHT] is that I'm more careful with my health because I have HIV, so I have to take care of my liver and if it's the way that I'm going to die soon—I have to stop… I'm more aware of my health. If my health becomes compromise if taking the hormonal therapy, I have to stop it or not take it. (Participant 2, age 29)

“I decided to have that HIV Test, when I found out that I'm positive about HIV, I decided to stop [FHT] because …it can be affect my medication, that's why doctor told me as well that you have to stop it because it's not good for yourself that you are taking the HIV medication and then you're taking that hormones, maybe it can affect your blood system. (Participant 8, age 25)

## Discussion

This paper provides an empirical account of perspectives and experiences regarding decisions to take FHT and ART medications among Filipina trans women living with HIV, with perspectives from their HIV providers demonstrating the challenges these women experience in navigating their health needs. Based on the thematic findings of our qualitative analysis, we present a working conceptual framework regarding gender-affirmative HIV care in Fig 1. Such a framework has not been previously proposed within the HIV or hormone treatment literature addressing the health of trans women. This framework captures both positive and negative dynamics of provider-patient encounters that influence trans women’s decisions for FHT with ART. It also depicts the affirmative versus non-affirmative rhetoric styles that can take place during these encounters, and how these styles may align or misalign with trans women’s gender affirmation goals—thereby influencing FHT and ART decisions. We discuss social and national policy-level implications for addressing the health needs of this highly vulnerable group in the context of HIV treatment settings.

HIV providers in this study expressed multiple clinical concerns, ranging from knowledge- and attitude-based concerns (i.e., not knowing about trans women’s health needs, viewing FHT as cosmetic) to infrastructural concerns (i.e., lack of workforce capacity/training, and dearth in research on FHT-ART interactions) regarding continuation of FHT while prescribing ART to trans women. Most providers did not view trans women’s FHT as important to health and would often prioritize ART over FHT. These views have been previously found in other studies where HIV providers perceived hormones as less important and were uncomfortable prescribing them [37–40]. As providers in our study prioritized ART over FHT, less focus was placed on actually meeting the expressed FHT needs of all trans women in our study; many providers actively dissuaded them from this treatment. These providers’ concerns and their prioritization of ART over FHT could be a reflection of their limited training and the lack of explicit policies around HIV and transgender health topics as curricular requirements. Currently, the Philippines’s public health and medical curricula do not include transgender health, either in or out of the context of HIV prevention and treatment [7, 41]. Moreover, although trans women are identified as a key population in the Philippines’ national HIV strategy plan [7], the plan does not explicitly set targets and policies to address trans women’s health needs distinctively from other key populations living with HIV, and does not describe the role of healthcare providers in delivering services to this important population. As such, our study underscores the need to improve HIV treatment delivery among trans women at the levels of public health policy and medical infrastructure in the Philippines. Our findings also support a need to prioritize trans women’s care explicitly in the national HIV strategic policy plan by setting specific goals for this community separately from other groups [7, 24]. Because knowledge,- attitudes, and infrastructural concerns appear to impact provider and trans women communications, future research should explore interventions targeting these aspects, both at the infrastructural- and provider-levels, as a means to inform future gender-affirmative approaches within the context of HIV care in the Philippines.

When communicating with trans women, providers have the opportunity to create an environment that is affirming to trans women’s health needs [39], including in the context of HIV-related and FHT services [13, 21]. Based on our findings, we characterized a gender-affirmative HIV care when providers utilize a rhetoric style that aligns with and supports the goals of taking FHT for trans women living with HIV. This rhetoric style affirms trans women’s goals such that it responds to and recognizes the importance of FHT’s functionality in supporting mental health benefits and self-confidence avoiding stigma, discrimination, and violence, as well as in affirming trans women’s gender identity and womanhood. It has been documented that allowing trans women to access gender-affirming services like FHT has multiple benefits including lower risk for unprotected sex with serodiscordant and unknown status partners, and lower street injection drug use and heavy substance use [13]. Provision of gender-affirmative care also improves mental health outcomes (e.g., lower depressive symptoms) among trans women [42], an outcome that is also known to optimize ART adherence in HIV treatment settings [43]. As such, future research for this population should examine how to optimally integrate FHT and other gender-affirming services (e.g., sex reassignment surgery), develop evidence-based health policies that support these services, as well as understand the multiple health benefits (i.e., mental health, violence prevention, HIV risk transmission reduction etc.) that this integration provides to trans women who are living with HIV in the Philippines.

Trans women in this study who experienced gender-affirmative HIV care reported taking medically monitored FHT and ART concurrently. This rhetoric style enabled providers to develop an affirming interaction with trans women, such that it allowed them to monitor both FHT and ART medications and their impact on trans women’s bodies, including proper and consistent laboratory services to check and ensure safety. This finding support recommendations that continued monitoring of FHT’s and ART’s impacts on the body is a best-practice for trans women living with HIV who desire to continue taking both medicines [12, 15, 22, 26]. Because monitoring requires multiple visits, providers expressing affirmative rhetoric could likely help retain trans women in care with their HIV provider [15, 44]. This approach is also consistent with principles of patient-centered practices (i.e., respecting patients’ preferences and responding appropriately to patient’s needs) in clinical settings [22, 45]. Given the retention challenges in the HIV care and treatment continuum [17, 46, 47], future research should examine ways to optimize the marketing and utilization of gender-affirmative HIV care approach to increase adherence and retention of trans women in the HIV care and treatment continuum.

Our study also reveals that trans women who experience a non-affirmative HIV care communication approach (e.g., being told to stop taking FHT) by their HIV providers reported facing difficult decisions to either not tell their provider about continuing to take FHT or follow their provider’s advice to stop taking FHT. Based on our findings, we characterized gender non-affirmative HIV care when providers use rhetoric that misaligns with trans women’s FHT goals, such that HIV treatment is more critical to living health and staying alive than FHT. Providers who used this rhetoric also cited that FHT is for cosmetic use, which has been documented before among providers who are unfamiliar with FHT and trans women’s health needs [37–40, 48]. This finding may have a historical precedent, such that FHT and other gender-affirming services like sex reassignment and facial feminization surgeries were once viewed as cosmetic commodities by insurance companies [49] and the scientific community [50–52]. As such, HIV providers in this study may have echoed this sentiment in their views.

FHT-ART outcomes arising from gender non-affirmative HIV care have various clinical implications. For instance, not fully disclosing FHT medication history to providers is a primary concern insofar as providers are not able to monitor how trans women’s bodies respond to both medications [26]. As such, withholding important information about FHT erases FHT from the purview of their providers, which could also inadvertently put trans women at risk of compromising their health [48, 53]. Similarly, the perception that HIV providers who use non-affirmative rhetoric style compels a choice between life versus death (i.e., taking ART only to live, and dying as a result of taking both medications) places trans women in risky situations where they are no longer receiving FHT-related health benefits and could cause poor mental health and exposure to physical violence [42, 54–56]. Interventions to improve mental health of trans women who are living with HIV and who face rejection or feel unsupported by their HIV providers are therefore highly needed. As an interim measure before implementing a large overhaul to train HIV providers in this area in the Philippines, HIV counselors or mental health providers in these health facilities should at least be made aware of these implications. Providers should be prepared to discuss how to facilitate proper patient communication and provide help to trans women who may comply with medical advice to cease FHT. These clinical outcomes resulting from non-affirmative care present real consequences for trans women, which must be addressed by HIV providers and mental health counselors in the Philippines.

Lastly, given that all trans women in our study reported currently taking ART meds, one FHT-ART outcome not observed but possible is prioritization of FHT over ART. The literature on FHT prioritization has previously been described before in other settings, noting that trans women are engaging in behaviors that affirm their trans identity at the expense of other health concerns like HIV (e.g., prioritizing taking FHT over ART) [12, 13, 20, 27]. This pattern of prioritization is likely because of the multiple benefits of FHT in improving quality of life [57] and mental health [58, 59]. This prioritization could include decisions such as partially taking ART (i.e., low ART adherence), completely discontinuing ART (i.e., no ART adherence), or not visiting an HIV provider (i.e., complete disengagement of HIV care). As such, trans women who decide to prioritize FHT over ART present critical clinical scenarios for the HIV treatment continuum that further add to the challenges in curbing the HIV epidemic and highlight the importance of integrating a gender-affirmative HIV care approach as part of the HIV response in the Philippines. Another possible scenario that is not depicted in this study’s qualitative data, but warrants future investigation, is exploring FHT-ART outcomes when both affirming and non-affirming rhetoric styles occur with a provider. For example, trans women might receive inconsistent styles of health communication and affirmative/non-affirmative messaging from providers across multiple visits, which might lead to further confusion, disengagement, and compromises to health.

### Limitations

This research has several limitations. First, these results do not describe the full range of outcomes concerning FHT and ART. Because none of our participants had chosen to drop out of HIV care as a result of their discussions about FHT and ART, we were unable to examine factors across narratives that may drive trans women living with HIV completely away from care. Second, we did not collect data on contextual factors about participants’ ART history (e.g., how long they have been living with HIV, when ART was started) and transition history (e.g., when they first began identifying as trans), which could have provided useful information in designing future interventions and determining the extent the results of this sample is extendable to the overall group of trans women in the Philippines. Third, the narratives collected via recruitment strategies may not represent all experiences of trans women living with HIV in the Philippines; other relevant venues where trans women can be found (e.g., online, support groups) were not recruitment sites in this study. Also, because these interviews were performed in English by US-based interviewers, legacies of colonialism may have created a dynamic producing social desirability bias in responding to questions [60–62]. Lastly, these interviews were done with trans women and providers in Metro Manila, where HIV services, knowledge about trans women, and awareness about HIV may be higher than in other cities and provinces in the Philippines [10]. As a result, these findings may not generalize to the experiences of trans women with HIV and their providers living outside the Metro Manila area.

## Conclusion

Taken together, this study provides a preliminary overview of a gender-affirmative HIV care framework based on the perspectives and experiences of Filipina trans women living with HIV and their HIV providers. To our knowledge, this is the first empirical study that describes such a framework for how various clinical outcomes regarding FHT and ART might arise from the affirmative vs. non-affirmative rhetoric use by HIV providers and its alignment or misalignment to trans women’s gender affirmation goals. Further research is needed to provide quantitative validation and elaborate on this preliminary framework, including: (a) exploring HIV provider-focused interventions to improve knowledge, attitudes, and motivation to interact with and attend to the health needs of trans women who are living with HIV, (b) examining ways for implementing gender-affirmative HIV care as a package that can be utilized to improve uptake, retention, and adherence to HIV care and treatment among trans women; and (c) understanding how to integrate FHT-ART co-prescribing practices to HIV care providers in the context of gender-affirmative services and the current infrastructure for HIV services in the Philippines. Along with research implications, these results suggest that there is a need for multi-level interventions that incorporate content relevant to addressing the inadequate gender-affirmative HIV care services for trans women in the Philippines. Such multifaceted interventions may include: 1) having policymakers and government agencies support policies around anti-discrimination specific to trans women in various settings, including healthcare facilities; 2) setting national targets for reducing gender-based violence and increasing gender-affirmatives services like FHT specific to trans women as part of the Philippine’s national HIV strategic plan; 3) including gender-affirmative HIV care in the medical and public health curricula and training programs for current and future health care providers; and 4) improving national HIV surveillance systems to accurately and reliably capture trans women’s HIV continuum of care as well as their gender affirmative services uptake. Along with the findings of this study, these research implications and recommendations support the call to integrate trans health across social and infrastructural public health and health policy solutions to address the HIV crisis in the Philippines.

## Supporting information

S1 Table Consolidated criteria for reporting qualitative research (COREQ) checklist. (PDF)
